# Effect of Periodontal Treatment on Metabolic Syndrome Parameters: A Systematic Review

**DOI:** 10.1111/odi.70018

**Published:** 2025-07-02

**Authors:** Anna Simonelli, Filippo Citterio, Fabio Falcone, Francesco D'Aiuto, Nicola Marco Sforza, Salvatore Corrao, Giorgio Sesti, Leonardo Trombelli

**Affiliations:** ^1^ Research Centre for the Study of Periodontal and Peri‐Implant Diseases University of Ferrara Ferrara Italy; ^2^ Operative Unit of Dentistry AUSL of Ferrara Ferrara Italy; ^3^ Private Practice in Monza Monza Italy; ^4^ Department of Clinical Medicine, Internal Medicine Unit National Relevance and High Specialization Hospital Trust ARNAS Civico, Di Cristina, Benfratelli Palermo Italy; ^5^ Department of Health Promotion Sciences, Maternal and Infant Care, Internal Medicine and Medical Specialties. [PROMISE] University of Palermo Palermo Italy; ^6^ Periodontology Unit UCL Eastman Dental Institute London UK; ^7^ Private Practice in Bologna Bologna Italy; ^8^ Department of Clinical and Molecular Medicine Sapienza University of Rome Rome Italy

**Keywords:** metabolic syndrome, non‐surgical periodontal therapy, periodontitis, systematic review, systemic inflammation

## Abstract

**Aim:**

To evaluate the effects of periodontal treatment on metabolic syndrome (MS) parameters and systemic inflammatory biomarker levels in patients affected by both MS and periodontitis.

**Methods:**

A systematic search was conducted to identify randomized controlled trials (RCTs) with a follow‐up ≥ 1 month comparing a more intense periodontal treatment (test intervention) to either a less intense or no periodontal treatment (control intervention). MS parameters such as triglycerides (TG), high‐density lipoprotein cholesterol (HDL), fasting blood glucose (FBG), glycated hemoglobin (HbA1c), systolic blood pressure (SBP), diastolic blood pressure (DBP), waist circumference (WC), and systemic inflammatory biomarkers (i.e., C‐reactive protein, CRP) were analyzed. Random‐effects meta‐regression analyses were conducted at 3‐ and 6‐month follow‐ups.

**Results:**

Four RCTs were included. Test intervention resulted in a statistically significant greater SBP reduction at 3 (WMD 1.48) and 6 (WMD 1.88) months compared to control group. A statistically significant difference between groups was observed for FBG at 6 months after treatment, favoring test intervention (WMD 0.61). No differences were observed for the other MS parameters and for CRP at both 3‐ and 6‐month follow‐up.

**Conclusions:**

Periodontal treatment seems to have beneficial effects on SBP and FBG in patients with MS and periodontitis at 3–6‐month follow‐ups.

**Trial Registration:**

This systematic review was registered under the protocol registration number CRD42024499854/PROSPERO

## 1 Introduction

Periodontitis is a common chronic, non‐communicable disease, characterized by a progressive destruction of soft and hard tissue keeping teeth in place (Papapanou et al. 2018). Pathogenesis of periodontitis is complex with several aspects not completely understood including a deranged host immune response, a heritable genetic susceptibility, environmental and behavioral factors, significantly contributing to disease onset and progression (Kinane and Mark Bartold 2007). Its primary features include the loss of periodontal tissue support, manifested through clinical attachment (CAL) and bone loss, presence of deepened periodontal pockets (PD) and gingival bleeding. When left untreated periodontitis inevitably will result in the loss of teeth impacting on the oral and overall health and quality of life (Uy et al. 2022).

Periodontitis is an often overlooked but relevant public health problem due to its high prevalence (GBD 2017 Oral Disorders Collaborators et al. 2020), an expensive and time‐consuming treatment, and its association with other chronic diseases related to premature mortality, such as diabetes, cardiovascular and respiratory diseases, cancer, and Alzheimer's disease (Chapple et al. 2013; Tonetti et al. 2013).

Metabolic syndrome (MS) is a clustering of risk factors for cardiovascular diseases and type 2 Diabetes Mellitus, including high blood pressure, dyslipidemia (i.e., high triglycerides levels and low‐density lipoprotein levels), high plasma glucose, and central obesity. According to the definition given by Alberti et al. (2009), a diagnosis of MS can be made when at least three of these components co‐exist. MS represents a serious health concern mainly due to the high pro‐inflammatory and pro‐thrombotic levels (Wilcox 2005; Grundy 2016) that increase two to fivefold the risk for developing cardiovascular disease and type 2 Diabetes Mellitus, respectively (Eckel et al. 2005; Alberti et al. 2006).

MS and periodontitis are linked by a bidirectional relationship (Nibali et al. 2013; Jepsen et al. 2020). Longitudinal studies with follow‐ups ranging from 3 to 17 years (Iwasaki et al. 2015; Furuta et al. 2016; Kaye et al. 2016) have shown that the persistence of MS is associated with a higher risk for tooth loss (Kaye et al. 2016) as well as worsening of periodontitis (Iwasaki et al. 2015; Kaye et al. 2016). On the other hand, a recent investigation conducted on Japanese adults followed up for 8 years demonstrated that individuals presenting worse periodontal health (periodontal pockets with PD ≥ 6 mm) are at significantly increased risk of developing MS, thus supporting the notion that periodontitis could be a risk factor for the development of MS (Kaye et al. 2016).

In patients with periodontitis, active periodontal treatment aims to reduce or eliminate gingival inflammation and periodontal pockets, thereby preventing tooth loss and preserving dental function (Loos and Needleman 2020). The initial therapeutic phase (i.e., Steps I and II) involves supragingival plaque removal and risk factor control, combined with subgingival plaque and calculus removal at sites exhibiting loss of periodontal support and pockets, with or without adjunctive antimicrobial therapy. If residual inflammation persists after Steps I and II, additional treatment (i.e., Step III) including nonsurgical re‐instrumentation or surgical correction of residual anatomical defects, may be considered (Sanz et al. 2020).

Besides the improvement in gingival health, treatment of periodontitis has been linked to systemic health benefits (Orlandi et al. 2021; Herrera et al. 2022) that are particularly evident in patients affected by severe forms of periodontitis when in association with one or more chronic non‐communicable diseases. In these patients, treatment of periodontitis has been demonstrated to substantially lower the overall systemic inflammatory burden (Orlandi et al. 2021) through the reduction of biomarkers such as the C‐reactive protein (CRP) (Offenbacher et al. 2009; Higashi et al. 2009; D'Aiuto et al. 2018) interleukin (IL) 6 (Higashi et al. 2009; D'Aiuto et al. 2018), and tumor necrosis factor alpha (TNF‐α) (Chen et al. 2012), and improve blood vessel function (Masi et al. 2018; Orlandi et al. 2021) at 6 and 12 months after treatment. Since systemic inflammation triggers insulin resistance (Demmer et al. 2012) via insulin antagonization and interference with lipid metabolism (Nesto 2004; King 2008), its reduction may improve the patient metabolic profile as assessed by fasting blood glucose (FBG) (Teeuw et al. 2010; D'Aiuto et al. 2018; Orlandi et al. 2021) and glycated hemoglobin (HbA1c) levels (Teeuw et al. 2010; D'Aiuto et al. 2018).

Over the last ten years experimental clinical evidence has been conducted on the effects of the treatment of periodontitis on MS (Acharya et al. 2010; López et al. 2012; Torumtay et al. 2016; Bizzarro et al. 2017; Montero et al. 2020; Doke et al. 2021; Milanesi et al. 2023). Although restricted to a limited number of clinical studies (Doke et al. 2021; Milanesi et al. 2023), there is some evidence of a potential beneficial effect of medical and dental treatment combined in patients affected by both periodontitis and MS. However, to date, there is no systematic appraisal of the available evidence on the impact of the treatment of periodontitis on MS and its parameters including systemic inflammation. Therefore, this review aimed to address the following focused questions: “*Does periodontal treatment have an influence on MS parameters*?” and “*Does periodontal treatment have an influence on the levels of systemic inflammatory biomarkers and pro‐inflammatory cytokines in patients affected by MS*?”

## 2 Materials and Methods

### 2.1 Standards of Reporting

The protocol of this systematic review (SR) followed the PRISMA (Preferred Reporting Items for Systematic Review and Meta‐Analyses) statement (Page et al. 2021). The review protocol was registered and allocated in the PROSPERO International Prospective Register of Systematic Reviews (id number: CRD42024499854).

### 2.2 Review Question

The investigation question was designed using the PICOTS acronym:

- Population (P)—Adult patients affected by Periodontitis, as defined according to the current (Tonetti et al. 2018) or previous case definitions criteria (Miller et al. 1994; Tonetti et al. 2005; Page and Eke 2007; Eke et al. 2012), and MS, defined according to the currently used criteria (The Examination Committee Of Criter 2002; Alberti et al. 2009; Grundy 2016);
- Intervention (I)—A least completion of Step I and II treatment phases (Sanz et al. 2020) with/without adjunctive use of systemic antibiotics;
- Comparison (C)—no treatment for periodontitis or treatment of periodontitis at lower intensity than the test group. A procedure was considered at “lower intensity” when less invasive (e.g., limited to supragingival plaque removal or not using of additional local/systemic medications) for the patient when compared to the intervention;
- Outcome (O)—MS parameters, including triglycerides (TG), high‐density lipoprotein cholesterol (HDL), FBG, HbA1c, systolic blood pressure (SBP), diastolic blood pressure (DBP), waist circumference (WC), as well as systemic inflammatory biomarkers;
- Time (T)—at least 1 month of follow‐up;
- Setting (S)—randomized clinical trials (RCTs).

### 2.3 Selection Criteria

Studies must have met the following inclusion criteria: (1) RCTs with at least 1 month of follow‐up; (2) studies with periodontal status assessment by applying full‐mouth examination; (3) studies with a clear case definition of periodontitis based on clinical and/or radiographic parameters; (4) studies involving patients with MS undergoing mechanical supra‐ and subgingival dental plaque removal with/without adjunctive use of systemic antibiotics (test group), compared with no treatment or a less intensive treatment of periodontitis (control group), and (5) studies in which a diagnosis of MS was set on the basis of currently accepted parameters, including hypertension, dyslipidemia, high plasma glucose, and central obesity.

The following studies were excluded: non‐randomized controlled trials, observational, and cohort prospective or retrospective studies; RCTs written in non‐Latin (Roman) alphabet. Multiple studies that evaluated the same sample were excluded, with only the longest follow‐up study being included.

### 2.4 Evidence Search Strategy

An electronic evidence search was performed on two electronic databases (Medline‐Pubmed and Embase) to identify potentially eligible articles published up to March the 4th 2024. Two independent reviewers (A.S. and F.C.) screened the titles and abstracts retrieved through the search process. The same reviewers selected full manuscripts of studies meeting the inclusion criteria, or those with insufficient data in the title and abstract to make a clear decision. Any disagreements between the reviewers were resolved by open discussion and consensus. If no consensus could be reached, a third author (LT) was consulted. The inter‐reviewer agreement (kappa correlation coefficient) of the screening and full‐text analysis phases was calculated.

A series of search terms combined with Boolean operators “AND” and “OR” was used. The following search string was developed:

- Pubmed

((Periodontal disease OR Periodontitis OR “Periodontitis” [Mesh] OR Scaling OR Root Planing OR Etiological Treatment OR “Dental Scaling”[Mesh] OR “Periodontal Debridement” [Mesh] OR “Root Planing”[Mesh] OR “Dental Prophylaxis”[Mesh])) AND (metabolic syndrome OR “Metabolic Syndrome”[Mesh] OR “waist circumference” [Mesh]) AND (Therapy/Narrow[filter]). Excel spreadsheets were created to record information pertaining to the decision to include or exclude each article.

- Embase

(scaling OR ‘root planing’ OR ‘dental scaling’/exp. OR ‘dental debridement’/exp. OR ‘root planing’/exp. OR ‘dental prophylaxis’/exp. OR ‘periodontitis’/exp. OR periodontitis OR ‘periodontal disease’/exp) AND (metabolic AND syndrome OR ‘waist circumference’) AND ‘randomized controlled trial’.

### 2.5 Data Collection Process and Data Items

A pilot spreadsheet was filled out by A.S. with data from the included articles and independently checked by F.C. and F.F. All the discrepancies that emerged were resolved by another adjudicator (L.T.).

The relevant methodological data and results with statistical measures were extracted from the articles (Table S1). All corresponding Authors of the included studies were contacted via email in cases where data were not available or not clearly described in the paper.

### 2.6 Risk of Bias Assessment

For the included RCTs, methodological quality assessment was performed using the Version 2 of the Cochrane tool for assessing risk of bias in randomized trials (RoB 2) (Sterne et al. 2019). One trained researcher (A.S.) addressed the five main domains for risk of bias: randomization process (D1), deviations from the intended interventions (D2), missing outcomes data (D3), measurement of the outcomes (D4), and selection of the reported results (D5). A risk of bias judgment (among “low risk of bias”, “some concerns”, or “high risk of bias”) was assigned to each domain (depending on the descriptions given for each field) and to the entire study. Assessments were then discussed among the reviewers to confirm agreement (L.T., F.C.).

### 2.7 Effect Measures and Synthesis Methods

STATA Version 18.0 (StataCorp. 2023. Stata Statistical Software: Release 18. College Station, TX: StataCorp LLC.) was used to perform all the analyses. Mean values and standard deviations derived from treatment and control groups for each study were used to provide an estimated standardized mean difference (SMD) relevant to the size of the intervention effect (i.e., the difference in means) and relative to the variability observed in that study. The meta‐analysis was performed if at least two studies were available for each comparison at various follow‐ups. An effect‐size meta‐analysis was performed. We computed the modified Glass statistics using the pooled sample standard deviation and Cochran's Q statistic. The pooled mean effect‐size estimate was calculated according to methods described by Hedges and Olikin (1985). According to the Glass method, statistics (standardized mean difference by pooled standard deviation) and 95% confidence intervals were calculated. Heterogeneity was investigated using the *I* ^2^ statistic with significance set at *p* < 0.05. The random effect model was used to perform the meta‐analysis independently of the heterogeneity. The forest plot was used to graphically summarize evidence from the various studies.

## 3 Results

### 3.1 Search and Screening

A total of 445 studies were identified and, after excluding duplicates and screening titles and abstracts, only 5 studies were considered potentially eligible for any quantitative analysis (Figure S1). The kappa value for inter‐rater agreement was 1.00 (95% CI: 1.000–1.000). Among the five eligible studies, the one by Lopez et al. (López et al. 2012) was also excluded since data related to MS parameters could not be either extracted from the manuscript (data were only reported in graphs and not in tables) or provided by the contacted authors. Additionally, the study by Bizzarro et al. (Bizzarro et al. 2017) encompassed a patient population broader than that defined by our inclusion and exclusion criteria. Consequently, the corresponding author was contacted to obtain data pertaining specifically to the MS sub‐population. As a result, four publications were pooled for meta‐analysis.

### 3.2 Study Characteristics

All studies were published between 2017 and 2023 and set in four different countries: one study was conducted in The Netherlands (Bizzarro et al. 2017), one in Spain (Montero et al. 2020), one in Japan (Doke et al. 2021) and one Brazil (Milanesi et al. 2023). Three trials were performed in a university setting, and 1 in a hospital setting (Doke et al. 2021). Overall, a total of 333 patients were evaluated (sample sizes ranging from 30 to 158 individuals) and followed up for one (Doke et al. 2021), three (Bizzarro et al. 2017; Montero et al. 2020; Doke et al. 2021; Milanesi et al. 2023), six (Bizzarro et al. 2017; Montero et al. 2020; Milanesi et al. 2023), and twelve months (Bizzarro et al. 2017).

In two studies (Montero et al. 2020; Milanesi et al. 2023), periodontitis was classified according to the criteria defined in the 2018 EFP/AAP classification (Tonetti et al. 2018), while the studies by Bizzarro et al. (2017) and Doke et al. (2021) used older case definitions criteria (i.e., Miller et al. (1994) and Tonetti et al. (2005), respectively). Great heterogeneity was also observed in the criteria used to diagnose MS: two studies used the classification proposed by Alberti et al. (2009), one study use the classification by Grundy (2008) while the study by Doke et al. (2021) used the Japanese waist circumference criteria of MS (The Examination Committee Of Criter 2002) (Table S1).

### 3.3 Interventions for Periodontitis and MS

In all studies, test groups received supra and subgingival scaling and root planning and oral hygiene instructions. In the studies by Bizzarro et al. (2017) and Montero et al. (2020) systemic antibiotics were prescribed in addition to subgingival debridement (amoxicillin 375 mg plus metronidazole 250 mg, 3 times/day for 7 days; azithromycin 500 mg, 1 time/day for 3 days, respectively). Intervention in control groups varied from no treatment (Doke et al. 2021; Milanesi et al. 2023), supragingival instrumentation plus adjunctive administration of a placebo (Montero et al. 2020) to standard supra and subgingival dental plaque removal (Bizzarro et al. 2017).

As for MS management during the experimental treatment phase, it ranged from no treatment (Bizzarro et al. 2017), unchanged treatment regimen (i.e., medical treatment remained unaltered during the study period) (Montero et al. 2020), dietary and exercise counseling (Doke et al. 2021; Milanesi et al. 2023), or adjustments in the treatment (if needed) (Milanesi et al. 2023) (Table S1).

### 3.4 Effect of Interventions on Periodontal Conditions

Overall, a wide heterogeneity among studies was observed for periodontal conditions at baseline in terms of % of sites with PD ≥ 4 mm [ranging from 15.1% (Doke et al. 2021) to 60.4% (Milanesi et al. 2023)], % of sites positive to BoP (BoP score; ranging from 20.0% (Doke et al. 2021) to 67.8% (Montero et al. 2020)) and mean PD [ranging from 2.9 mm (Milanesi et al. 2023) to 4.1 mm (Bizzarro et al. 2017)] (Table S2).

After treatment, an overall greater improvement was observed in test compared to control groups for all evaluated parameters. In particular, a statistically significant decrease in the percentage of sites with PD ≥ 4 mm was reported at 1, 3, and 6 months in test groups, with a mean PD ranging from 2.3 to 2.8 mm (Montero et al. 2020; Doke et al. 2021; Milanesi et al. 2023). These results were paralleled by a statistically significant decrease in BoP scores. In contrast, in the control group, the reduction in BoP score and sites with PD ≥ 4 mm was smaller and less consistent over observation intervals (Montero et al. 2020; Doke et al. 2021; Milanesi et al. 2023) (Table S2).

### 3.5 Effect of Interventions on MS Parameters and Inflammatory Biomarkers

Two studies (Montero et al. 2020; Milanesi et al. 2023) reported a statistically significant decrease in FBG 3 months after treatment of periodontitis in both test and control groups, without significant between‐group differences. The study by Montero et al. (2020) observed a reduced FBG also at the 6‐month visit in both groups. Similarly, a statistically significant decrease in WC from baseline to 1 – (Doke et al. 2021), 3 – (Doke et al. 2021) and 6 – (Milanesi et al. 2023) month follow‐up was observed in both test and control groups, without significant between‐group differences. In one study (Milanesi et al. 2023), a reduction in WC was observed only in the control group at 3months.

A statistically significant between‐group difference was reported at 3 months for HbA1c% (Montero et al. 2020), the magnitude of the reduction being greater in the test group. In one study, a statistically significant reduction in SBP and DBP was observed at the 1‐month follow‐up in both test and control group (Doke et al. 2021). For SBP, a reduction was reported at 3 months by Doke et al. (2021) in the test and control groups with no significant between‐group differences. At the same follow‐up, a statistically significant greater reduction in SBP was reported by Montero et al. (2020) only in the test group. For DBP, a statistically significant intra‐group decrease was observed in the control group at the 3‐month follow‐up (Doke et al. 2021). No intra‐ or between‐group differences in HDL cholesterol and TG were observed in either the control or test groups at follow‐up visits.

For CRP levels, a statistically significant greater reduction was recorded only in the test group of one study (Montero et al. 2020) whilst no differences between interventions at 3 and 6 months were reported in the other study (Milanesi et al. 2023).

The effect of interventions on MS parameters and inflammatory biomarkers is summarized in Table S3.

Patient‐related outcomes were not reported in any of the included studies. Only Montero et al. (Montero et al. 2020) reported adverse events, documenting two occurrences considered unrelated to the study intervention.

### 3.6 Risk of Bias

Among the included RCTs, four were judged to be a low risk of bias while one was considered to be at high risk of bias. The source of the high risk of bias was due to an appropriate analysis used to estimate the effect of assignment to intervention (Figure S2).

### 3.7 Meta‐Analysis

**FIGURE 1:**
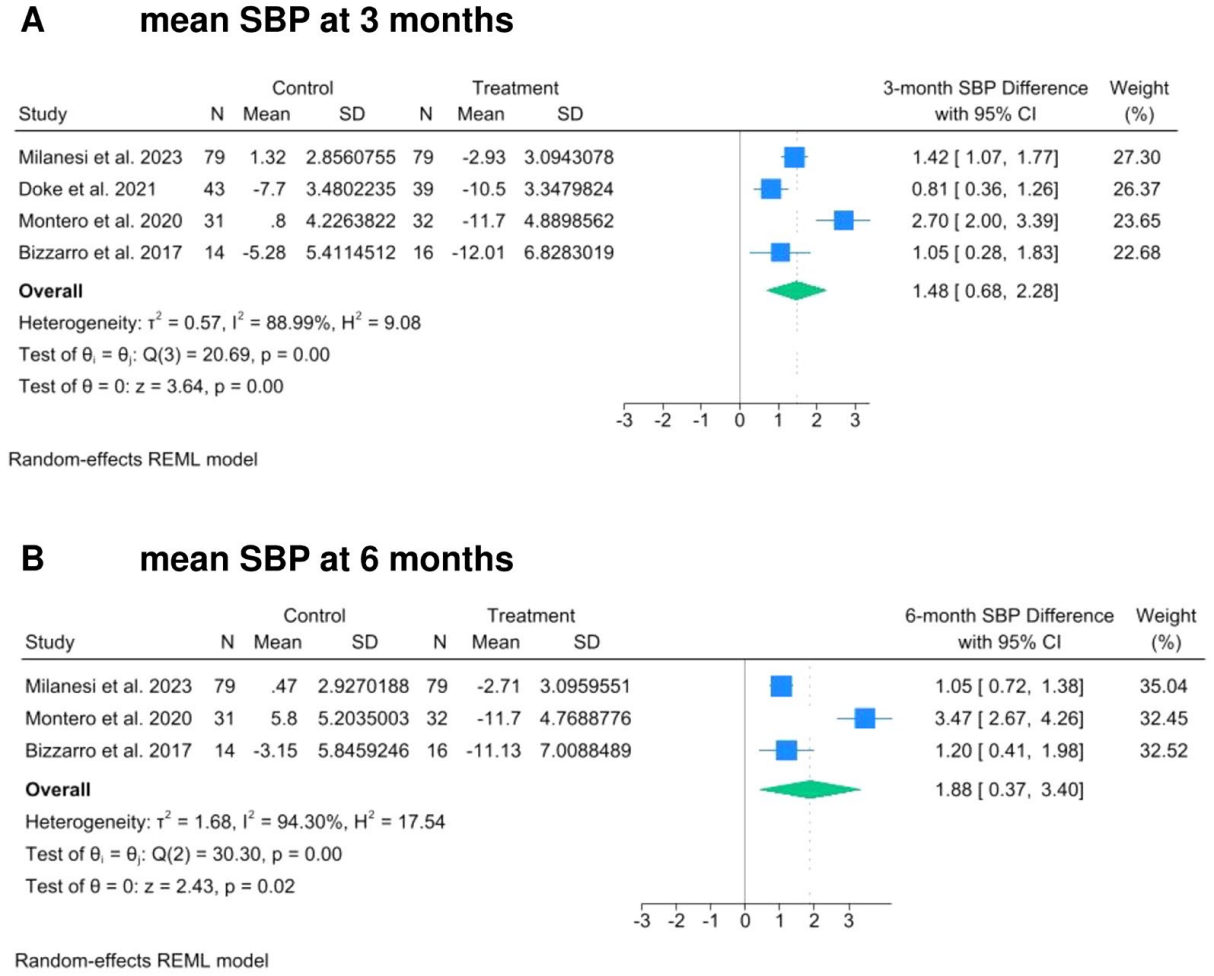
Effect of the treatment of periodontitis on Systolic Blood pressure (SBP) level (mmHg). The Forest Plot summarizes the mean SBP level changes at 3‐ (A) and 6‐ (B) months after non‐surgical periodontal treatment.

**FIGURE 2:**
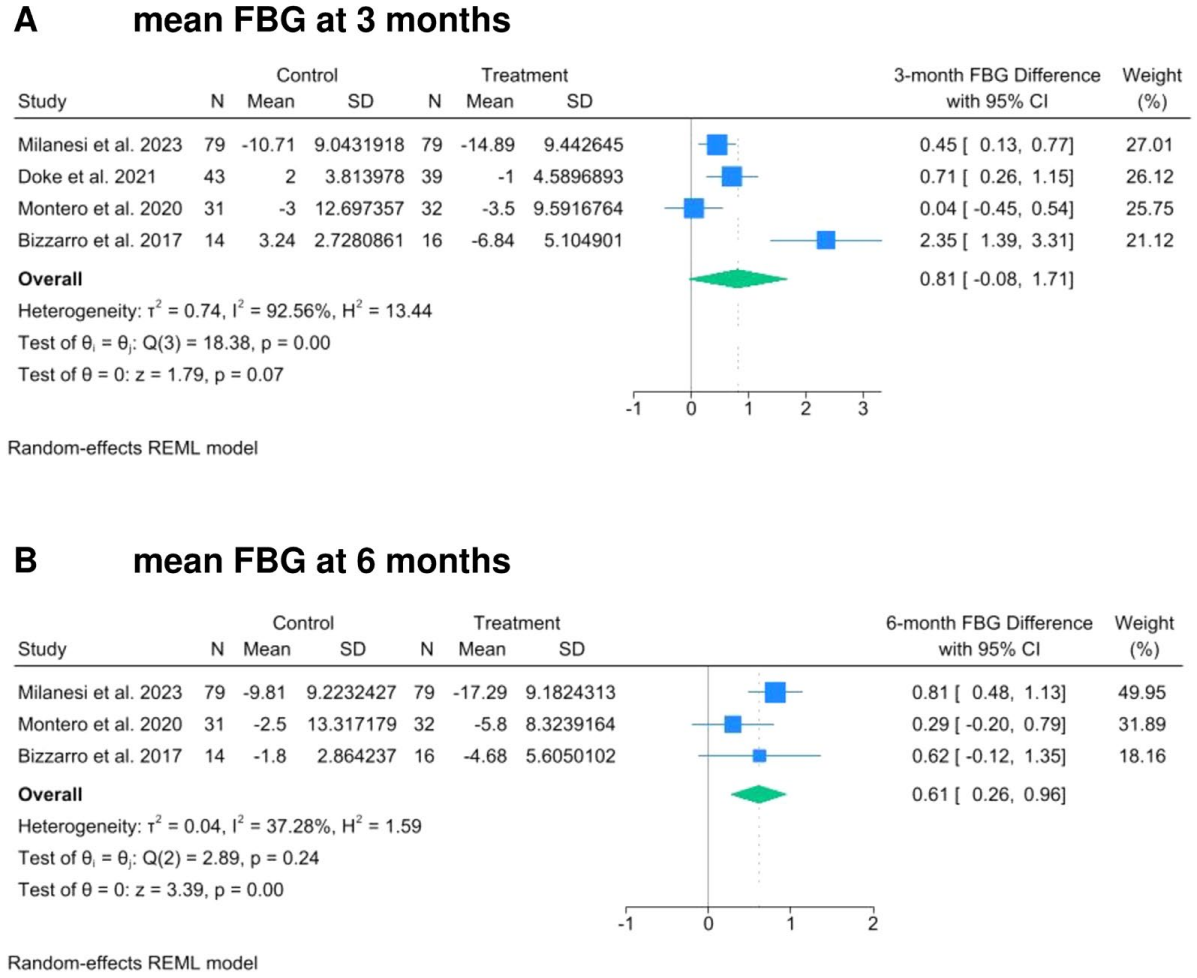
Effect of the treatment of periodontitis on Fasting Blood Glucose (FBG) level (mg/dL). The Forest Plot summarizes the mean FBG level changes at 3‐ (A) and 6‐ (B) months after non‐surgical periodontal treatment.

Random‐effects meta‐regression analyses were conducted using data recorded at 3‐ and 6‐month follow‐up (Figures 1, 2 and S3–S8). Test intervention resulted in a statistically significant greater SBP reduction at 3 (WMD 1.48, 95% Cl: 0.68 to 2.28, *p* = 0.00, *I* ^2^ = 88.99%) and 6 (WMD 1.88, 95% Cl: 0.37 to 3.40, *p* = 0.02, *I* ^2^ = 94.30%) months compared to control group. A statistically significant difference between groups was observed for FBG only at 6 months after treatment, favoring the test intervention (WMD 0.61, 95% Cl: 0.26 to 0.96, *p* = 0.00, *I* ^2^ = 37.38%). At both 3‐ and 6‐ month follow‐up, no significant differences were observed for MS parameters such as DBP (WMD 0.37, 95% Cl: −1.08 to 1.81, *p* = 0.62, *I* ^2^ = 96.99% and WMD 1.55, 95% Cl: −0.35 to 3.46, *p* = 0.11, *I* ^2^ = 96.49%, respectively), HbA1c% (WMD 0.91, 95% Cl: −0.14 to 1.96, *p* = 0.09, *I* ^2^ = 94.08% and WMD 0.80, 95% Cl: −0.68 to 2.27, *p* = 0.29, *I* ^2^ = 95.32%, respectively), HDL (WMD 0.29, 95% Cl: −0.07 to 0.66, *p* = 0.12, *I* ^2^ = 58.90% and WMD 0.44, 95% Cl: −0.20 to 1.09, *p* = 0.18, *I* ^2^ = 80.95%, respectively), TG (WMD 0.19, 95% Cl: −1.00 to 1.39, *p* = 0.75, *I* ^2^ = 95.77% and WMD 0.65, 95% Cl: −0.17 to 1.46, *p* = 0.12, *I* ^2^ = 87.17%, respectively), WC (WMD 0.16, 95% Cl: −0.25 to 0.58, *p* = 0.44, *I* ^2^ = 67.56% and WMD 0.21, 95% Cl: −0.04 to 0.46, *p* = 0.10, *I* ^2^ = 0.00%, respectively) as well as for CRP (WMD 0.57, 95% Cl: −2.34 to 3.48, *p* = 0.70, *I* ^2^ = 98.55% and WMD 0.40, 95% Cl: −2.47 to 3.28, *p* = 0.78, *I* ^2^ = 98.58%, respectively) (Figures S3–S8).

No differences were observed for MS parameters such as DBP, HbA1c%, HDL, TG, WC as well as for CRP at both 3‐ and 6‐month follow‐up.

## 4 Discussion

This review suggested that there is evidence, although limited, on a positive effect of step I‐II of periodontal treatment in reducing FBG (at 6 months after treatment) and SBP (at 3 and 6 months after treatment) in patients suffering from MS and periodontitis. Even if some studies reported some reductions in markers of systemic inflammation, this review did not identify sufficient evidence to support these findings.

Whilst there is consistent observational evidence suggesting a moderate association between periodontitis and MS, probably mediated by the elevated levels of systemic inflammation shared by both diseases, only a few interventional studies have addressed the question of whether this association is causal in nature (i.e., periodontitis contributing to the cardio‐metabolic derangement observed in patients with multiple cardiovascular risk factors).

Treatment of periodontitis aims at downregulating gingival inflammation (i.e., BoP score < 30% of sites) and achieving shallow gum pockets (i.e., PD < 4 mm), both conditions being associated with stable periodontal health and lower risk for tooth loss over time (Tonetti et al. 2018; Sanz et al. 2020). The present SR included studies evaluating non‐surgical periodontal interventions with test groups receiving more intensive treatment than control groups. Treatment in test groups included risk factor control and supra/subgingival plaque and calculus removal, with (Bizzarro et al. 2017; Montero et al. 2020) or without (Doke et al. 2021; Milanesi et al. 2023) adjunctive systemic antibiotics. Treatment in control groups ranged from no treatment (Doke et al. 2021; Milanesi et al. 2023) to supragingival professional plaque removal (Montero et al. 2020) or subgingival instrumentation without antibiotics (Bizzarro et al. 2017). Test interventions positively impacted on the prevalence of sites with PD ≥ 4 mm and BoP score which showed a significantly greater decrease in test compared to control group. Pocket reduction resulted particularly effective in the studies by Montero et al. (2020) and Doke et al. (2021) where the prevalence of sites with PD ≥ 4 mm decreased below 14% at all follow‐up visits.

One of the results observed in this review was the positive effect of test interventions on arterial blood pressure levels, with reduction of the SBP values at 3 and 6 months. However, the difference in DBP between test and control group did not reach statistical significance at all observation intervals. Interestingly, these results are in line with recent evidence suggesting that SBP profile is more consistently associated with periodontitis than DBP, both in terms of magnitude and strength of the association (Muñoz Aguilera et al. 2020; Del Pinto et al. 2020). On this regard, recent RCTs conducted on patients with different systemic health conditions (e.g., individuals affected by hypertension, prehypertension, or diabetes) (Zhou et al. 2017; D'Aiuto et al. 2018) reported SBP reductions ranging from 3 mmHg to 11.1 mmHg after step I and II of periodontal therapy whereas DBP values showed less evident changes (from −0.8 to 8.3 mmHg). In our analysis, a mean difference of 1.88 mmHg in SBP [95% CI: 0.37–3.40] was observed at the 6‐month follow‐up between more intensive and less intensive periodontal treatments. While this difference may appear modest, it has been shown to carry meaningful clinical implications for vascular disease risk. Evidence from a large‐scale meta‐analysis involving nearly one million adults without prior vascular disease demonstrated that a 2 mmHg reduction in usual SBP is associated with a 10% decrease in stroke mortality and a 7% decrease in ischemic heart disease mortality among middle‐aged individuals (Lewington et al. 2002). Although these estimates are based on “usual” blood pressure rather than “single point” measurements—as used in the studies included in the present review—they nonetheless suggest that even small reductions in SBP may yield notable benefits in terms of vascular risk. Additionally, for the correct interpretation of our data, it must be also noted that other factors beyond the reduced periodontal inflammation could have contributed to the observed SBP reductions. Among these, it should be noted that in 2 out of 4 studies included in the present systematic review (Doke et al. 2021; Milanesi et al. 2023) patients received medicine prescriptions, adjustments of preexisting medical therapies as well as counseling for diet and physical activity.

The present meta‐analysis also confirmed the benefit of test interventions on FBG that significantly improved at the 6‐month follow‐up with a mean difference of 0.61 mg/dL (95% CI: 0.26–0.96, *p* = 0.00) compared to less intensive interventions. Considering the positive continuous association between FBG and cardiovascular risk highlighted by several authors and supported by biological principles that sustain how abnormal glucose metabolism impairs normal endothelial function, accelerates atherosclerotic plaque formation, and contributes to plaque rupture and thrombosis, the present finding assumes a relevance, especially in terms of prevention that finds in lifestyle interventions a more sustainable and preferable intervention compared to pharmacological interventions (Knowler et al. 2002).

The present meta‐analysis also confirmed the beneficial effect of the test interventions on FBG, which showed a significant improvement at the 6‐month follow‐up, with a mean difference of 0.61 mg/dL (95% CI: 0.26–0.96, *p* = 0.00) compared to less intensive interventions. Considering the well‐established positive continuous association between FBG levels and cardiovascular risk (Avendano and Mackenbach 2006; Danaei et al. 2006)—supported by mechanistic evidence indicating that impaired glucose metabolism compromises endothelial function, promotes atherosclerotic plaque development, and increases the risk of plaque rupture and thrombosis (Park et al. 2013)—this finding is particularly relevant in the context of cardiovascular prevention strategies prioritizing lifestyle‐based approaches over pharmacological treatment (Tuomilehto et al. 2001).

Additionally, it is noteworthy to underline that these results were paralleled by improvements also in HbA1c% (difference of 0.91% and 0.80% at 3 and 6 months follow‐up, respectively), that although not statistically significant, are in line with data from previous systematic reviews reporting HbA1c reductions ranging from 0.27% to 0.48% at 3–4 months after non‐surgical periodontal treatment (Corbella et al. 2013; Li et al. 2015).

In the present review, inconclusive evidence was retrieved on the effects of the treatment of periodontitis on other metabolic markers (i.e., HDL, TG, WC) as well as those of systemic inflammation (i.e., CRP) with two studies (Montero et al. 2020; Milanesi et al. 2023) reporting some differences between test and control interventions, while the other two studies did not observe any difference at follow‐up (Bizzarro et al. 2017; Doke et al. 2021). This finding is in contrast with other studies excluded from the present review, where individuals with MS showed substantial improvements in the levels CRP (Acharya et al. 2010; López et al. 2012; Torumtay et al. 2016), IL‐6 (Torumtay et al. 2016), TG (Acharya et al. 2010; Torumtay et al. 2016) and HDL (Acharya et al. 2010) after treatment of periodontitis. This observed discrepancy may be attributable to the substantial heterogeneity in both test and control interventions across the included studies. One notable factor potentially influencing the lack of consistent CRP reduction is the variability in step II treatment protocols. For instance, subgingival instrumentation combined with systemic antibiotics led to significant reductions in CRP (Montero et al. 2020), whereas subgingival instrumentation alone did not produce similar effects (Milanesi et al. 2023). Furthermore, the management of MS in the included studies often involved a range of pharmacological and lifestyle interventions, which may have independently affected systemic inflammation, thus confounding the specific impact of periodontal therapy.

### 4.1 Implications for Practice

The present SR confirmed that the treatment of periodontitis may have a positive effect on some MS cardio‐metabolic components and may be, therefore, considered as a novel non‐pharmacological intervention to be implemented in the MS management. Therefore, this finding calls for identifying periodontal care needs in patients with MS as a result of a closer collaboration between medical care colleagues and oral health professionals as an integral part of a multidisciplinary management of MS. Oral health screening tools should be implemented in medical practice and at‐risk individuals should be signposted to appropriate dental/periodontal care services to improve overall general health. Similarly oral health professionals could play a role in addressing the importance of managing common cardio‐metabolic risk factors in patients with MS either for opportunistic screening or informing of the impact of systemic inflammation and management of cardio‐metabolic risk in the general population.

### 4.2 Implications for Research

This review highlighted the scarcity of properly designed interventional trials addressing the potential benefit of the treatment of periodontitis on common cardio‐metabolic risk factors in patients without established chronic co‐morbidities like diabetes or cardiovascular diseases. Larger and better designed intervention trials should be performed to expand on the initial evidence identified in this review of a potential benefit in managing periodontal inflammation on established cardio‐metabolic risk factors such as SBP and glucose levels. This review also highlighted the need to address common risk factors in patients with oral health needs, hence suggesting that more health‐service research is needed to identify novel opportunities for dental health professionals in the management of common chronic disorders.

### 4.3 Study Limitations

The results of the present SR must be interpreted with caution in view of some important limitations. The first one is represented by the limited number of eligible studies included in the final qualitative and quantitative analyses. The paucity of the included investigations has inevitably affected the reliability of the results. Moreover, one study (Doke et al. 2021) was classified as “high” risk of bias since the analysis used to estimate treatment effect was not considered appropriate. Although possibly overstating the intervention effectiveness, this study was not excluded from the meta‐analysis. A robust and comprehensive methodology could only account in part for this limitation. Data heterogeneity related to the high variability in the diagnostic criteria used for MS (see Table S3) as well as on the great number of therapeutic approaches used in both MS and the treatment of periodontitis surely impacted on the results of this review. Finally, it was not possible to account for additional outcomes (i.e., adverse events after treatment or impact of the therapy on patient quality of life/reported outcomes) as none of the included studies recorded this information.

## 5 Conclusions

Within the limits of the present SR, there is some evidence to suggest that the treatment of periodontitis can improve common cardio‐metabolic risk factors (i.e., decrease in arterial blood pressure and improvements in the glycemic control) in patients with MS. Oral health promotion should become integral part of common risk factors management of the general public.

## Author Contributions

**Anna Simonelli:** writing – original draft, investigation. **Filippo Citterio:** investigation. **Fabio Falcone:** formal analysis, investigation. **Francesco D'Aiuto:** writing – original draft, validation. **Nicola Marco Sforza:** conceptualization. **Salvatore Corrao:** formal analysis. **Giorgio Sesti:** conceptualization. **Leonardo Trombelli:** conceptualization, writing – original draft.

## Conflicts of Interest

The authors declare no conflicts of interest.

## Supporting information


FIGURE S1–S8.

TABLE S1–S3.

## Data Availability

The data that support the findings of this study are available from the corresponding author upon reasonable request.
