# Supplementary material for: Effect of Periodontal Treatment on Metabolic Syndrome Parameters: A Systematic Review
Source: Oral Dis. 2025 Jul 2;31(12):3272–81. doi: 10.1111/odi.70018 (PMC12989050; doi:10.1111/odi.70018)

**Effect of periodontal treatment on metabolic syndrome parameters.**

**A systematic review.**

- **Supplementary figures-**

**SUPPLEMENTARY FIGURES**

**Figure S1.** Flowchart of the study selection process.


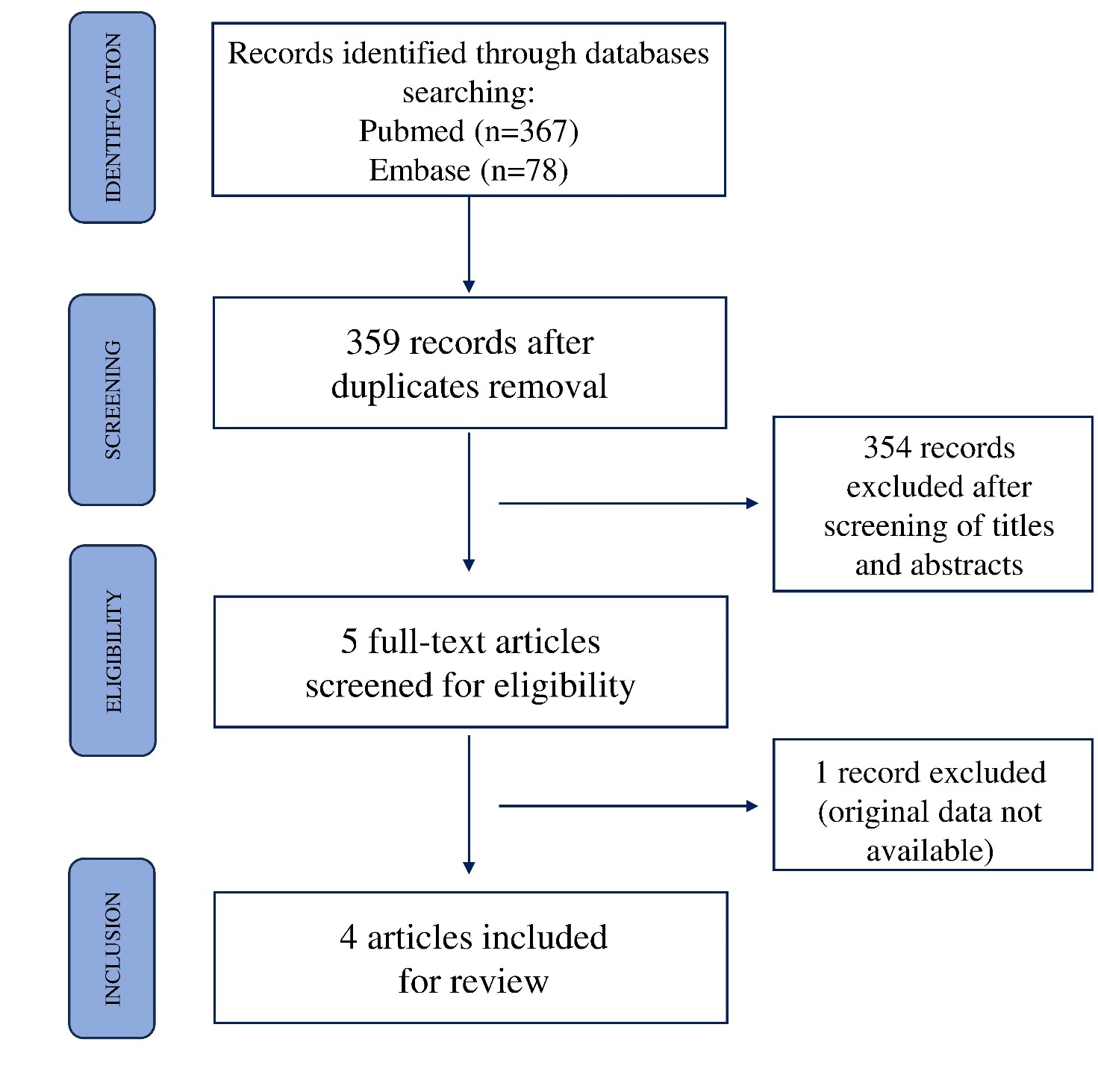


**Figure S2**. Risk of bias assessment for all included studies**.**


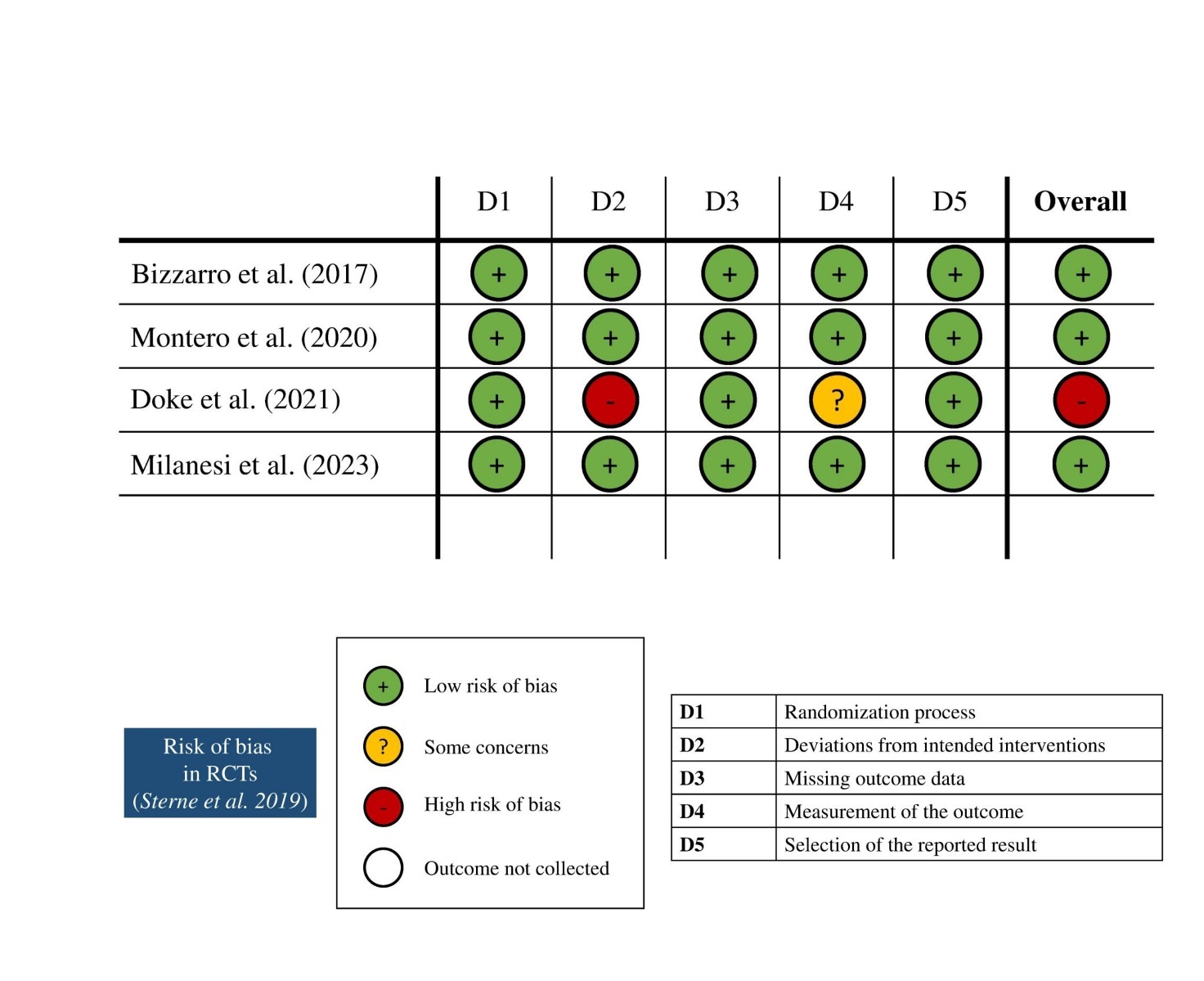


**Figure S3.** Effect of the treatment of periodontitis on Diastolic Blood pressure (DBP) level (mmHg). The Forest Plot summarizes the mean SBP level changes at 3- (A) and 6- (B) months after non-surgical periodontal treatment.


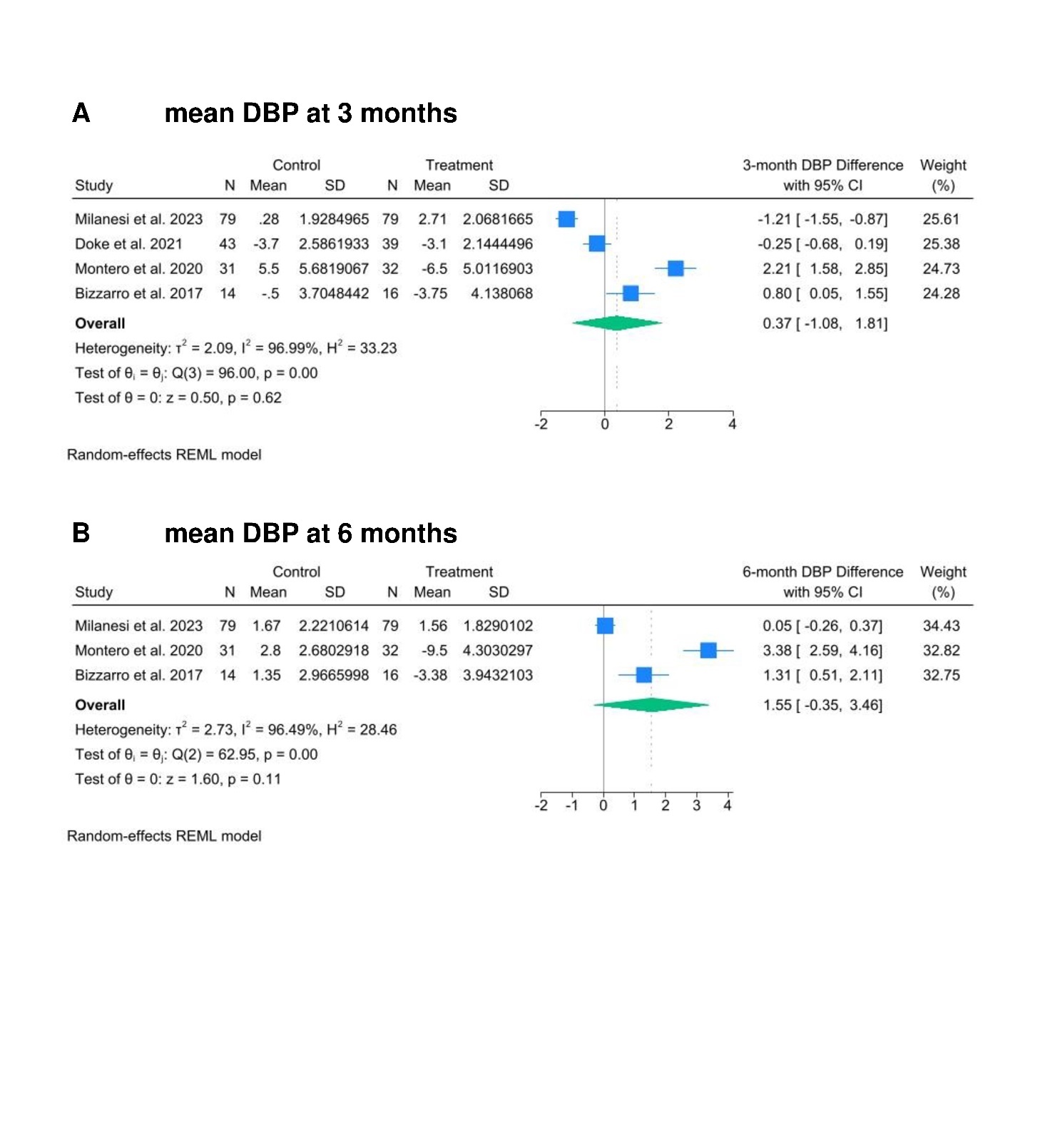


**Figure S4.** Effect of the treatment of periodontitis on glycated hemoglobin (HbA1c) level (%). The Forest Plot summarizes the mean HbA1c% level changes at 3- (A) and 6- (B) months after non-surgical periodontal treatment.


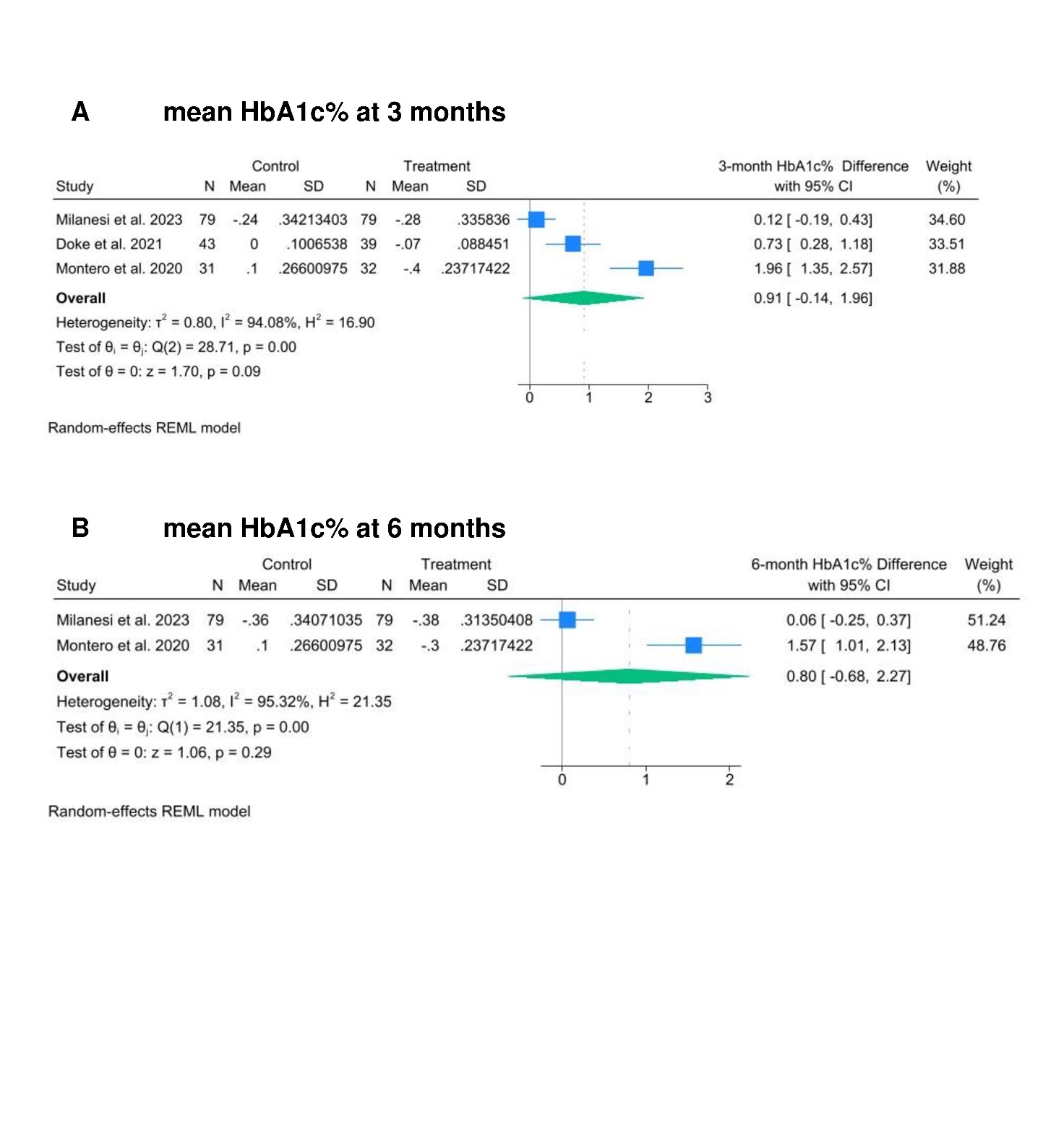


**Figure S5.** Effect of the treatment of periodontitis on high-density lipoprotein cholesterol (HDL) level (mg/dL). The Forest Plot summarizes the mean HDL level changes at 3- (A) and 6- (B) months after non-surgical periodontal treatment.


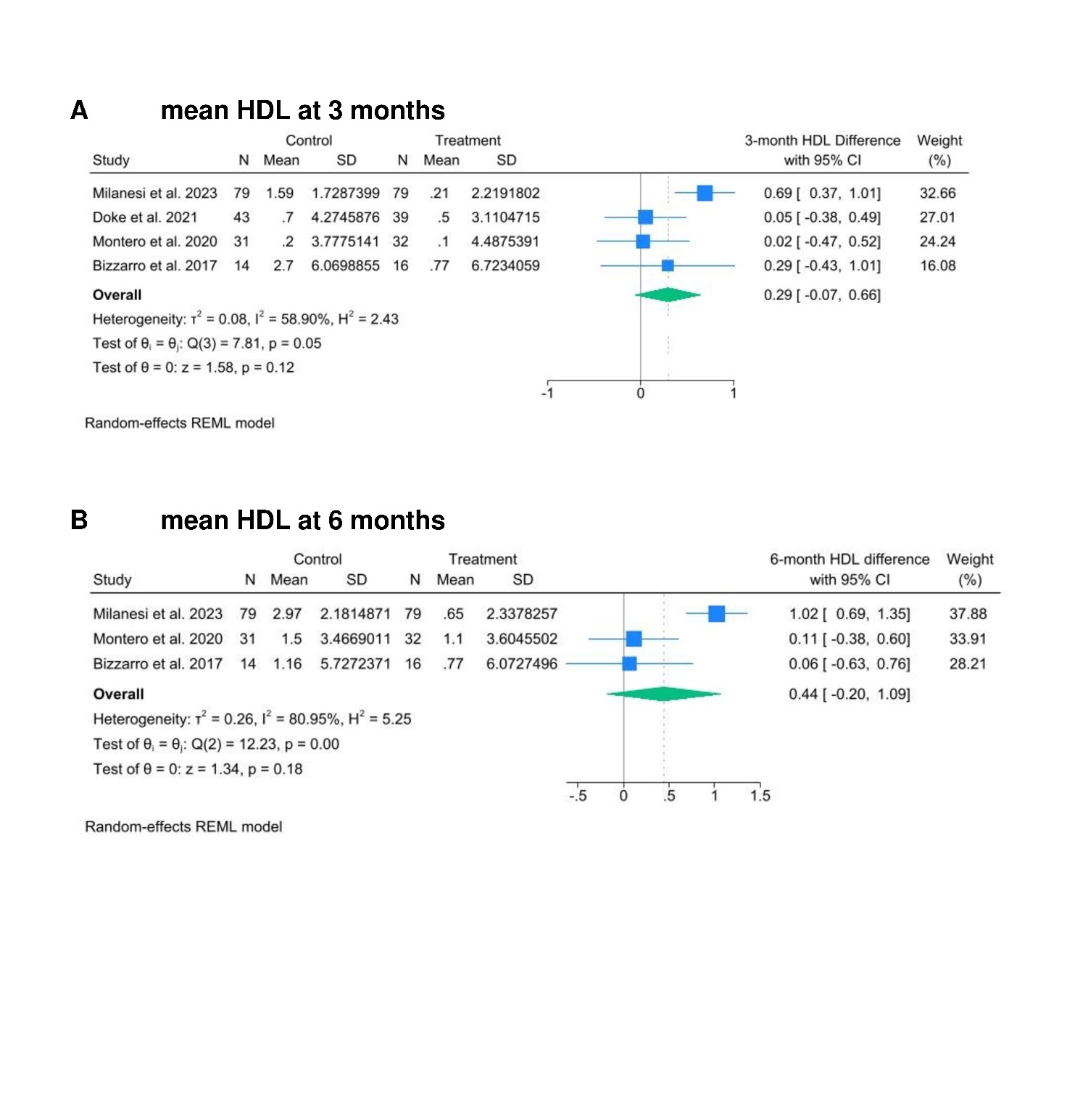


**Figure S6.** Effect of the treatment of periodontitis on triglyceride (TG) level (mg/dL). The Forest Plot summarizes the mean TG level changes at 3- (A) and 6- (B) months after non-surgical periodontal treatment.


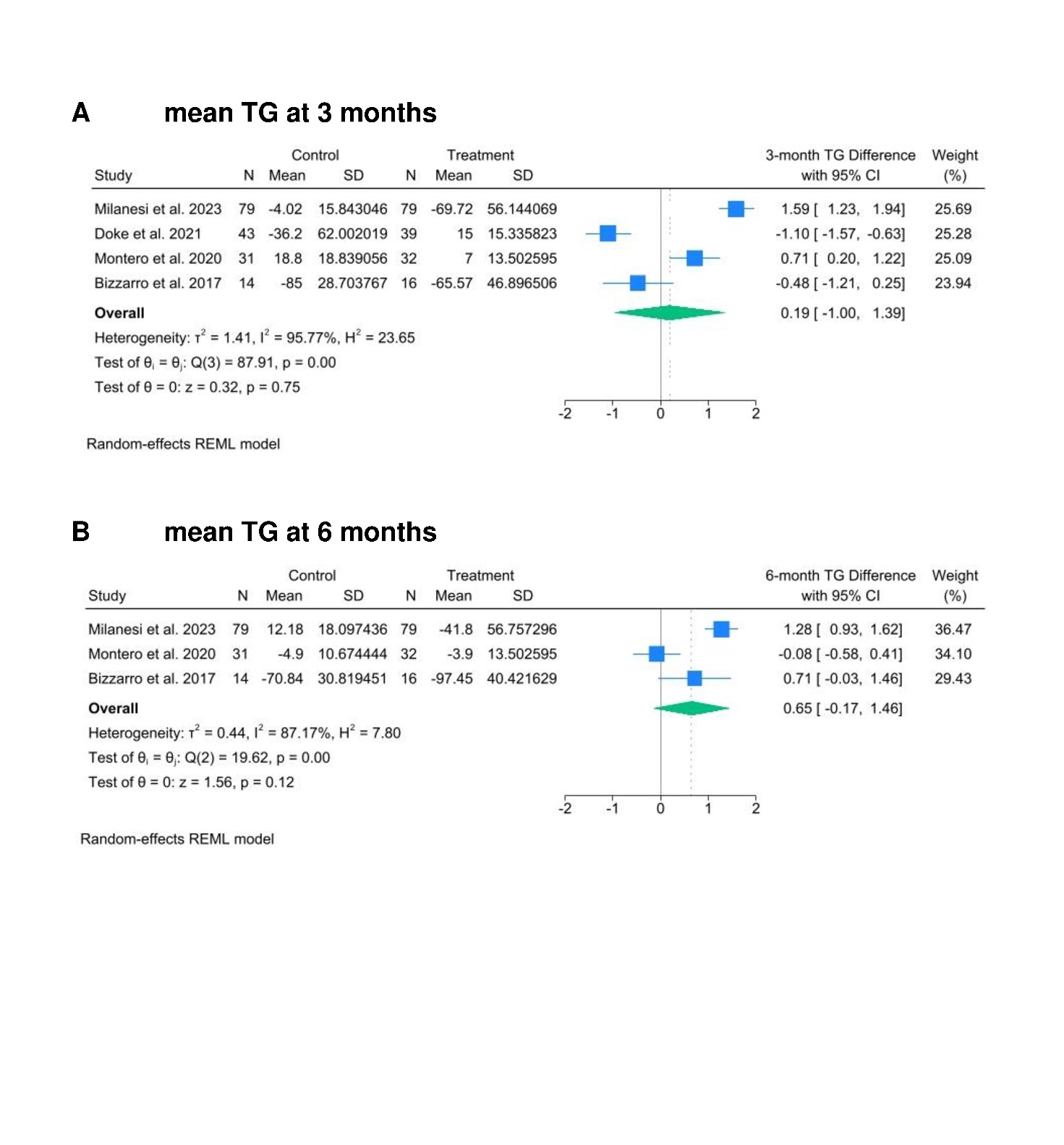


**Figure S7.** Effect of the treatment of periodontitis on waist circumference (WC) (cm). The Forest Plot summarizes the mean WC changes at 3- (A) and 6- (B) months after non-surgical periodontal treatment.


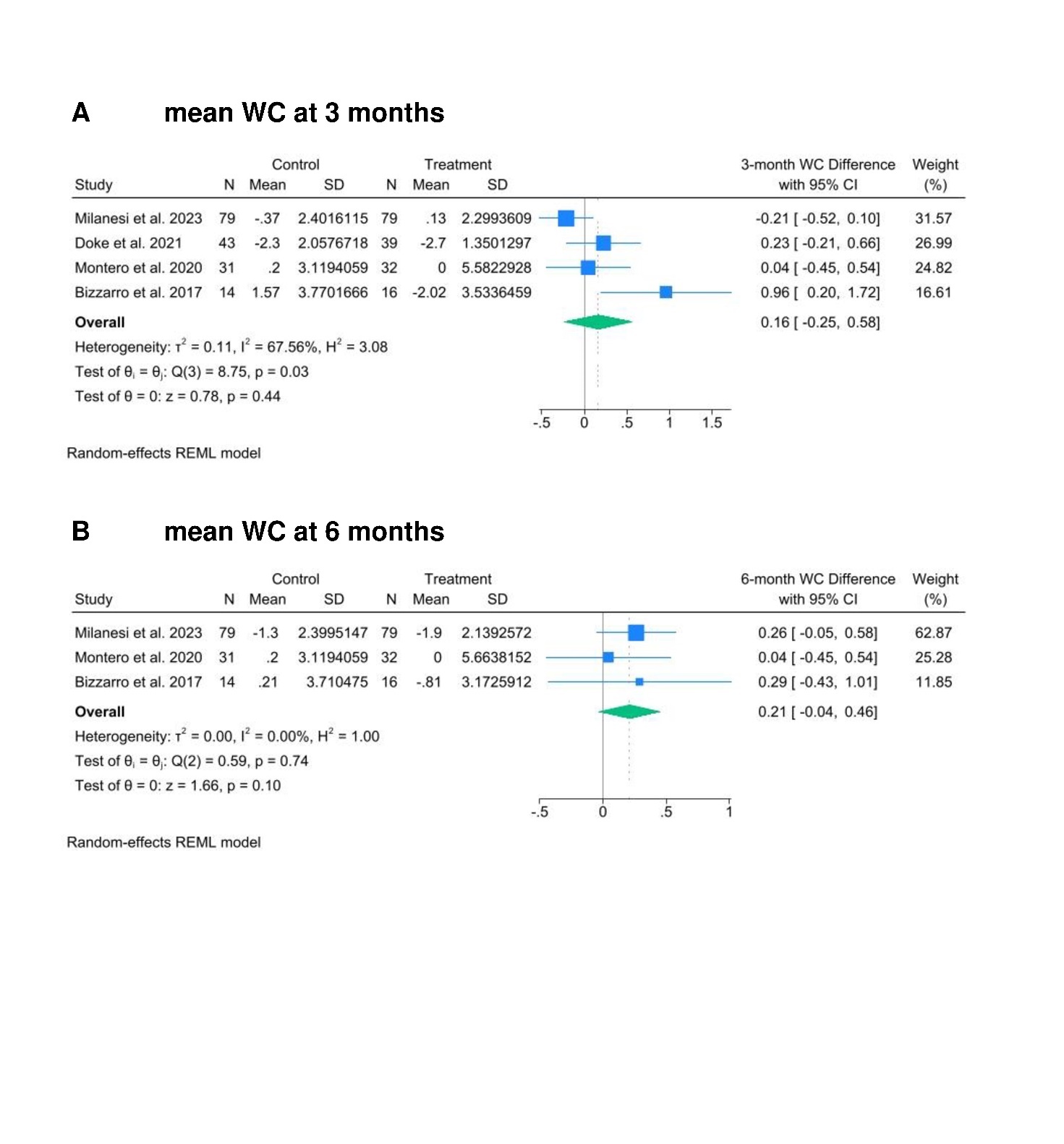


**Figure S8.** Effect of the treatment of periodontitis on C-reactive protein (CRP) levels (mg/L). The Forest Plot summarizes the mean CRP levels changes at 3- (A) and 6- (B) months after non-surgical periodontal treatment.


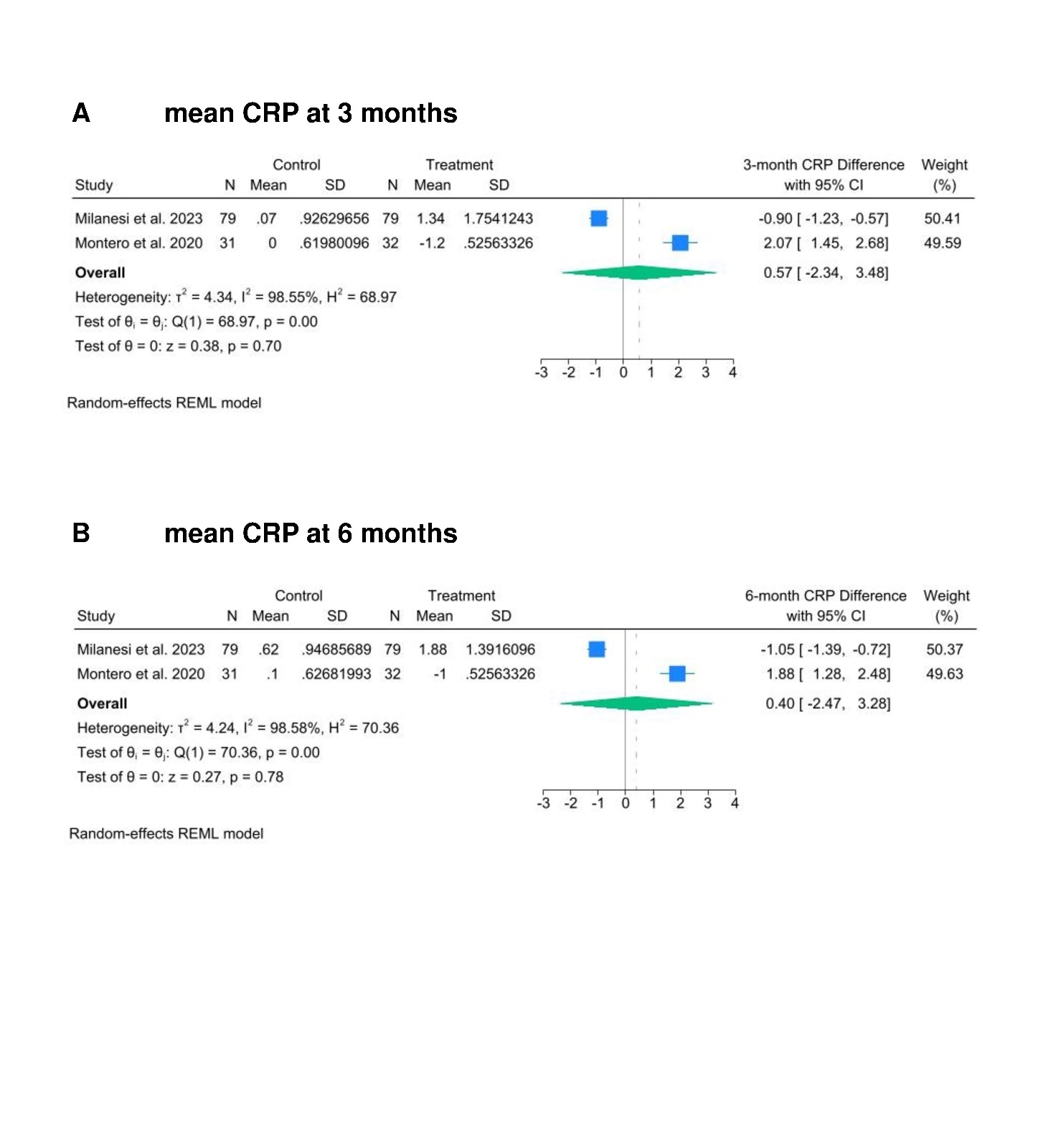

Supplement: Supplementary file 1 — FIGURE S1–S8. [file ODI-31-3272-s002.docx]
