# Supplementary material for: Effect of Periodontal Treatment on Metabolic Syndrome Parameters: A Systematic Review
Source: Oral Dis. 2025 Jul 2;31(12):3272–81. doi: 10.1111/odi.70018 (PMC12989050; doi:10.1111/odi.70018)
Supplement: Supplementary file 2 — TABLE S1–S3. [file ODI-31-3272-s001.docx]

**Effect of periodontal treatment on metabolic syndrome parameters.**

**A systematic review.**

- **Supplementary tables-**

**SUPPLEMENTARY TABLES**

**TABLE S1:** Main characteristics of the studies included in the review.

| **First author (year)** | **Country** | **Setting/**  **follow-ups** | **Study**  **Population**  **(n)** | **Diagnostic criteria for P** | **Diagnostic criteria for MS** | **MS Treatment**  **(yes/no)**  **Effectiveness of MS treatment** | **Type (intensity) and effectiveness of Periodontal treatment (yes/no)** |
| --- | --- | --- | --- | --- | --- | --- | --- |
| **Bizzarro 2017** | The Netherlands | University setting  3-, 6- and 12-month follow-ups | 110 patients of which 30 MS patients were included in the meta-analysis | Diagnosis definition:  Tonetti & Claffey 2005  Definition of P based on BoP, PD, REC and CAL values | Grundy et al. 2008 | No treatment for MS | *Test group*  *(n=16)*  Basic periodontal therapy (BTP) plus amoxicillin 375 mg and metronidazole 250 mg (both assumed 3 times/day for 7 days)  *Control group*  *(n=14)*  BTP without systemic antibiotic  BTP consisted of non-surgical periodontal treatment performed in 3 appointments within 1 week followed by  a 3-monthly maintenance program for 1 year. |
| **Montero 2020** | Spain | University setting  3- and 6- month follow-ups | 63 patients  (all affected by severe P and MS) | Diagnosis definition:  Tonetti et al. 2017  Definition of P based on BoP, PI, GI and PD and CAL values | Alberti et al. 2009 | Patients were treated for MS and their medical treatment remained unchanged during the study period | *Test group*  *(n=32 ITT analysis)*  Intensive periodontal treatment based on two  sessions of SRP plus adjunctive administration of a systemic antibiotic  (azithromycin 500 mg for 3 days), administered from the last session of SRP.  *Control group*  *(n=31 ITT analysis)*  Minimal periodontal treatment based on two  sessions of supragingival professional mechanical plaque removal,  without subgingival instrumentation. Placebo was added. |
| **Doke**  **2021** | Japan | Hospital setting  1- and 3-month follow-ups | 112 patients  (all affected by light-moderate P and MS) | Periodontitis definition:  Modified CPI  Definition of P based on PD values | Patients included in the study if positive to the Japanese waist circumference criteria of MS | Patients received dietary and exercise guidance, which consisted of the Total Fitness Analysis System and a video- programmed dietary and exercise lecture. | *Test group*  (n= 56 *ITT analysis*)  non-surgical periodontal treatment  *Control group*  (n= 56 *ITT analysis*)  no treatment (delayed) |
| **Milanesi 2023** | Brasil | University Hospital setting  3- and 6- month follow-ups | 158 patients  (all affected by moderate/severe P and MS) | Diagnosis definition:  Tonetti et al. 2017  Definition of P based on BoP, PD and CAL values | Alberti et al. 2009 | Patients were treated for MS and adjustments in the medical treatment were performed if needed.  Counselling for diet and exercises was given | *Test group*  *(n=79 ITT analysis)*  Non-surgical periodontal  treatment in 4 weekly sessions, consisting of SRP with manual and ultrasonic instruments. Personalized oral hygiene instructions were given.  Bi-weekly maintenance  appointments scheduled up to 3-month follow-up and monthly appointments up to 6-month follow-up.  *Control group*  *(n=79 ITT analysis)*  No treatment (delayed) |

Abbreviations: BoP: Bleeding on Probing; CAL: Clinical Attachment Level; CPI: Community Periodontal Index; ITT: Intention to-treat; GI: Gingival Index; MS: Metabolic Syndrome; P: periodontitis; PD: Pocket Depth; PI: Plaque Index; SRP: Scaling and Root Planning; REC: Gingival Recession.

**TABLE S2:** Periodontal parameters at baseline and at follow-ups in test and control groups, as reported in the included studies.

| **PD≥4mm (% of sites)** | | | | | | | | | | | | |
| --- | --- | --- | --- | --- | --- | --- | --- | --- | --- | --- | --- | --- |
|  | **BASELINE** | | | **1 month** | | | **3 months** | | | **6 months** | | |
|  | control | test | *p** | control | test | *p** | control | test | *p** | control | test | *p** |
| **Montero et al. 2020** | 59.7±19.0^†^ | 55.3±22.0^†^ | *0.427* | *---* | *---* | *---* | 41.2±23.0^†^ | 13.9±12.0^†^ | *<0.001* | 43.9±21.0^†^ | 11.2±10.0^†^ | *<0.001* |
| *p*** |  |  |  |  |  |  | *<0.05* | *<0.05* |  | *<0.05* | *<0.05* |  |
| **Doke et al. 2021** | 15.1±14.9^†^ | 19.1±16.3^†^ | *0.243* | 15.8±16.0^†^ | 11.3±10.0^†^ | *0.000* | 16.0±16.5^†^ | 10.4±10.2^†^ | *0.000* | --- | --- | --- |
| *p*** |  |  |  | *1.000* | *0.000* |  | *1.000* | *0.000* |  | *---* | *---* | *---* |
| **Milanesi et al.2023** | 57.7±27.55^§^ | 60.4±25.77^§^ | *0.52* | *---* | *---* | *---* | 51.9±28.61^§^ | 30.8±23.9^§^ | *<0.0001* | 48.5±28.53^§^ | 25.6±23.28^§^ | *<0.0001* |
| *p*** |  |  |  |  |  |  | *ns* | *<0.001* |  | *<0.05* | *<0.001* |  |
| **BoP (% of sites)** | | | | | | | | | | | | |
|  | **BASELINE** | | | **1 month** | | | **3 months** | | | **6 months** | | |
|  | control | test | *p** | control | test | *p** | control | test | *p** | control | test | *p** |
| **Montero et al. 2020** | 67.8±20.0^†^ | 59.8±20.0^†^ | *0.139* | *---* | *---* | *---* | 48.4±19.2^†^ | 24.9±21.0^†^ | *<0.001* | 51.6±18.0^†^ | 20.5±11.0^†^ | *<0.001* |
| *p*** |  |  |  |  |  |  | *<0.05* | *<0.05* |  | *<0.05* | *<0.05* |  |
| **Doke et al. 2021** | 20.0±18.4^†^ | 25.3±21.4^†^ | *0.235* | 19.4±19.2^†^ | 14.3±11.5^†^ | *0.001* | 18.7±18.7^†^ | 12.9±12.9^†^ | *0.002* | *---* | *---* | *---* |
| *p*** |  |  |  | *1.000* | *0.000* |  | *1.000* | *0.000* |  |  |  |  |
| **Milanesi et al.2023** | 58.7±25.4^§^ | 58.0±24.44^§^ | *0.86* | *---* | *---* | *---* | 51.9±24.61^§^ | 22.4±13.50^§^ | *<0.0001* | 50.7±26.39^§^ | 19.6±10.22^§^ | *<0.0001* |
| *p*** |  |  |  |  |  |  | *<0.001* | *<0.001* |  | *<0.001* | *<0.001* |  |
| **Mean PD (mm)** | | | | | | | | | | | | |
|  | **BASELINE** | | | **3 month** | | | **6 months** | | | **12 months** | | |
|  | control | test | *p** | control | test | *p** | control | test | *p** | control | test | *p** |
| **Bizzarro et al. 2017** | 3.79±0.59^†^ | 4.16±0.66^†^ | *na* | 2.80±0.52^†^ | 2.84±0.37^†^ | *na* | 2.85±0.47^†^ | 2.83±0.51^†^ | *na* | 2.83±0.57^†^ | 2.83±0.51^†^ | *na* |
|  |  |  |  |  |  |  |  |  |  |  |  |  |
| **Montero et al. 2020** | 3.8±0.9^†^ | 3.7±0.9^†^ | *0.747* | 3.5±0.7^†^ | 2.7±0.3^†^ | *<0.001* | 3.5±0.6^†^ | 2.6±0.3^†^ | *<0.001* | *---* | *---* | *---* |
| *p*** |  |  |  | *ns* | *<0.05* |  | *ns* | *<0.05* |  |  |  |  |
| **Milanesi et al.2023** | 2.95±0.79^§^ | 3.02±0.71^§^ | *0.59* | 2.80±0.71^§^ | 2.33±0.35^§^ | *<0.0001* | 2.79±0.79^§^ | 2.27±0.35^§^ | *<0.0001* | *---* | *---* | *---* |
| *p*** |  |  |  | *<0.001* | *<0.001* |  | *<0.001* | *<0.001* |  |  |  |  |

Abbreviations: BoP: Bleeding on Probing; na: not available; ns: not significant; PD: Pocket Depth.

* *inter-group comparison*

*** intra-group comparison when compared to baseline*

^§^ *data reported as mean (± standard error)*

† *data reported as mean (± standard deviation)*

**TABLE S3:** Clinical parameters related to MS and inflammatory biomarkers at baseline and follow-ups in test and control groups, as reported in the included studies.

| **FBG** | | | | | | | | | | | | | | | |
| --- | --- | --- | --- | --- | --- | --- | --- | --- | --- | --- | --- | --- | --- | --- | --- |
|  | **BASELINE** | | | **1 month** | | | **3 months** | | | **6 months** | | | **12 months** | | |
|  | control | test | *p** | control | test | *p** | control | test | *p** | control | test | *p** | control | test | *p** |
| **Bizzarro et al. 2017** | 5.83±0.38^†^  (mmol/L) | 5.97±0.97^†^  (mmol/L) | na | --- | --- | --- | 6.01±0.45^†^ | 5.59±0.46^†^ | na | 5.73±0.49^†^ | 5.71±0.69^†^ | na | 5.90±0.52^†^ | 5.50±1.22^†^ | na |
| *p*** |  |  |  |  |  |  | na | na |  | na | na |  | na | na |  |
| **Montero et al. 2020** | 133.0±51.7^†^  (mg/dl) | 128.6±30.3^†^  (mg/dl) | *0.500* | *---* | *---* | *---* | 130.0±8.8^§^ | 123.3±7.9^§^ | *0.112* | 130.5±9.7^§^ | 121.0±6.3^§^ | *0.336* | *---* | *---* | *---* |
| *p*** |  |  |  |  |  |  | *<0.05* | *<0.05* |  | *<0.05* | *<0.05* |  |  |  |  |
| **Doke et al. 2021** | 96.2±11.4^†^  (mg/dl) | 97.2±24.9^†^  (mg/dl) | na | 101.2±17.2^†^ | 96.4±16.2^†^ | *0.098* | 98.2±21.2^†^ | 96.2±16.1^†^ | *0.42* | --- | --- | --- | --- | --- | --- |
| *p*** |  |  |  | *0.170* | *1.000* |  | *1.000* | *1.000* |  |  |  |  |  |  |  |
| **Milanesi et al.2023** | 136.93±60.69^§^  (mg/dl) | 137.37±67.08^§^  (mg/dl) | *0.95* | --- | --- | --- | 126.22±52.70^§^ | 122.48±50.44^§^ | *0.74* | 127.12±55.11^§^ | 120.08±46.49^§^ | *0.40* | --- | --- | --- |
| *p*** |  |  |  |  |  |  | *0.03* | *0.01* |  | *0.26* | *0.89* |  |  |  |  |
| **HbA1c (%)** | | | | | | | | | | | | | | | |
|  | **BASELINE** | | | **1 month** | | | **3 months** | | | **6 months** | | | **12 months** | | |
|  | control | test | *p** | control | test | *p** | control | test | *p** | control | test | *p** | control | test | *p** |
| **Montero et al. 2020** | 6.0±1.0^†^ | 6.3±1.2^†^ | *0.328* | --- | --- | --- | 6.1±0.2^§^ | 5.9±0.1^§^ | *0.013* | 6.1±0.2^§^ | 6.0±0.1^§^ | *0.110* | --- | --- | --- |
| *p*** |  |  |  |  |  |  | *ns* | *<0.05* |  | *ns* | *<0.05* |  |  | | |
| **Doke et al. 2021** | 5.6±0.4^†^ | 5.6±0.4^†^ | na | 5.6±0.4^†^ | 5.5±0.3^†^ | *0.18* | 5.6±0.5^†^ | 5.53±0.4^†^ | *0.28* | --- | --- | --- | --- | --- | --- |
| *p*** |  |  |  | *0.850* | *0.300* |  | *1.000* | *0.750* |  |  |  |  |  |  |  |
| **Milanesi et al.2023** | 7.15±2.35^§^ | 7.20±2.26^§^ | *0.22* | --- | --- | --- | 6.91±1.93^§^ | 6.92±1.95^§^ | *0.53* | 6.79±1.91^§^ | 6.82±1.63^§^ | *0.77* | --- | --- | --- |
| *p*** |  |  |  |  |  |  | *0.19* | *0.91* |  | *0.13* | *0.96* |  |  |  |  |
| **HDL cholesterol** | | | | | | | | | | | | | | | |
|  | **BASELINE** | | | **1 month** | | | **3 months** | | | **6 months** | | | **12 months** | | |
|  | control | test | *p** | control | test | *p** | control | test | *p** | control | test | *p** | control | test | *p** |
| **Bizzarro et al. 2017** | 1.16±0.40^†^  (mmol/L) | 1.19±0.41^†^  (mmol/L) | na | --- | --- | --- | 1.23±0.45^†^ | 1.21±0.54^†^ | na | 1.19±0.41^†^ | 1.21±0.45^†^ |  | 1.18±0.42^†^ | 1.15±0.38^†^ | na |
|  | na | na |  |  |  |  | na | na |  | na | na |  | na | na |  |
| **Montero et al. 2020** | 46.9±12.4^†^  (mg/dl) | 46.1±13.3^†^  (mg/dl) | *0.858* | --- | --- | --- | 47.1±3.1^§^ | 46.2±3.8^§^ | *0.858* | 48.4±2.7^§^ | 47.2±2.7^§^ | *0.097* | --- | --- | --- |
| *p*** |  |  |  |  |  |  | *ns* | *ns* |  | *ns* | *ns* |  |  |  |  |
| **Doke et al. 2021** | 64.9±19.9^†^  (mg/dl) | 58.9±12.7^†^  (mg/dl) | *na* | 63.5±17.3^†^ | 60±13.6^†^ | *0.2* | 65.6±18.8^†^ | 59.4±15.2^†^ | *0.94* | --- | --- | --- | --- | --- | --- |
| *p*** |  |  |  | *0.650* | *1.000* |  | *1.000* | *1.000* |  |  |  |  |  |  |  |
| **Milanesi et al.2023** | 41.97±10.28^§^  (mg/dl) | 45.10±14.20^§^  (mg/dl) | *0.22* | --- | --- | --- | 43.56±11.42^§^ | 45.31±13.69^§^ | *0.53* | 44.94±16.44^§^ | 45.75±15.17^§^ | *0.78* | --- | --- | --- |
| *p*** |  |  |  |  |  |  | *0.19* | *0.91* |  | *0.14* | *0.97* |  |  |  |  |
| **Triglyceride** | | | | | | | | | | | | | | | |
|  | **BASELINE** | | | **1 month** | | | **3 months** | | | **6 months** | | | **12 months** | | |
|  | control | test | *p** | control | test | *p** | control | test | *p** | control | test | *p** |  |  |  |
| **Bizzarro et al. 2017** | 2.72±0.76^†^  (mmol/L) | 2.54±1.36^†^  (mmol/L) | na | --- | --- | --- | 1.76±1.01^†^ | 1.80±1.54^†^ | na | 1.92±1.13^†^ | 1.44±1.07^†^ | na | 2.02±0.97^†^ | 1.74±1.26^†^ | na |
| *p*** |  |  |  |  |  |  | na | na |  | na | na |  | na | na |  |
| **Montero et al. 2020** | 136.6±42.5^†^  (mg/dl) | 129.5±52.3^†^  (mg/dl) | *0.582* | --- | --- | --- | 155.4±17.5^§^ | 136.5±9.7^§^ | *0.984* | 131.7±8.3^§^ | 125.6±9.7^§^ | *0.895* | --- | --- | --- |
| *p*** |  |  |  |  |  |  | *ns* | *ns* |  | *ns* | *ns* |  |  |  |  |
| **Doke et al. 2021** | 188.6±383.9^†^  (mg/dl) | 146.5±58.5^†^  (mg/dl) | na | 150.2±100.4^†^ | 140.1±70.3^†^ | *0.66* | 152.4±127.5^†^ | 161.5±77.9^†^ | *0.28* | --- | --- | --- | --- | --- | --- |
| *p*** |  |  |  | *1.000* | *1.000* |  | *1.000* | *0.720* |  |  |  |  |  |  |  |
| **Milanesi et al.2023** | 179.41±88.24^§^  (mg/dl) | 238.16±491.29^§^  (mg/dl) | *0.85* | --- | --- | --- | 175.39±109.74^§^ | 168.44±87.49^§^ | *0.79* | 191.59±134.49^§^ | 196.36±114.56^§^ |  | --- | --- | --- |
| *p*** |  |  |  |  |  |  | *0.36* | *0.38* |  | *0.89* | *0.44* |  |  |  |  |
| **CRP (mg/L)** | | | | | | | | | | | | | | | |
|  | **BASELINE** | | | **1 month** | | | **3 months** | | | **6 months** | | | **12 months** | | |
|  | control | test | *p** | control | test | *p** | control | test | *p** | control | test | *p** |  |  |  |
| **Montero et al. 2020** | 3.9±3.4^†^ | 3.9±2.9^†^ | *0.831* | --- | --- | --- | 3.9±0.6^§^ | 2.7±0.4^§^ | *0.001* | 4.0±0.8^§^ | 2.9±0.4^§^ | *0.004* | --- | --- | --- |
| *p*** |  |  |  |  |  |  | *ns* | *<0.05* |  | *ns* | *<0.05* |  |  |  |  |
| **Milanesi et al.2023** | 5.27±5.68^§^ | 5.79±7.14^§^ | 0.29 | --- | --- | --- | 5.34±5.96^§^ | 7.13±13.86^§^ | *0.48* | 5.89±6.21^§^ | 7.67±10.10^§^ | *0.39* | --- | --- | --- |
| *p*** |  |  |  |  |  |  | *0.49* | *0.23* |  | *0.92* | *0.73* |  |  |  |  |
| **SBP (mmHg)** | | | | | | | | | | | | | | | |
|  | **BASELINE** | | | **1 month** | | | **3 months** | | | **6 months** | | | **12 months** | | |
|  | control | test | *p** | control | test | *p** | control | test | *p** | control | test | *p** | control | test | *p** |
| **Bizzarro et al. 2017** | 147.07±15.49^†^ | 148.44±20.57^†^ | na | --- | --- | --- | 141.79±13.94^†^ | 136.43±16.20^†^ | na | 143.92±16.51^†^ | 137.31±17.39^†^ | na | 143.92±16.51^†^ | 137.31±15.45^†^ | na |
| *p*** |  |  |  |  |  |  | na | na |  | na | na |  | na | na |  |
| **Montero et al. 2020** | 138.6±18.3^†^ | 148.1±21.5^†^ | *0.112* | --- | --- | --- | 139.4±2.7^§^ | 136.4±3.0^§^ | *0.008* | 144.4±4.1^§^ | 136.4±2.8^§^ | *0.574* | --- | --- | --- |
| *p*** |  |  |  |  |  |  | *ns* | *<0.05* |  | *ns* | *ns* |  |  |  |  |
| **Doke et al. 2021** | 136.7±17^†^ | 140.9±16.4^†^ | *na* | 126.6±13.9^†^ | 130.4±13.3^†^ | *0.47* | 129±14.5^†^ | 130.4±13.9^†^ | *0.6* | --- | --- | --- | --- | --- | --- |
| *p*** |  |  |  | *0.000* | *0.000* |  | *0.000* | *0.001* |  |  |  |  |  |  |  |
| **Milanesi et al.2023** | 138.27±16.71^§^ | 139.86±18.74^§^ | *0.64* | --- | --- | --- | 139.59±19.11^§^ | 136.93±20.13^§^ | *0.36* | 138.74±19.94^§^ | 137.15±20.15^§^ | *0.56* | --- | --- | --- |
| *p*** |  |  |  |  |  |  | *0.92* | *0.29* |  | *1.00* | *0.37* |  |  |  |  |
| **DBP (mmHg)** | | | | | | | | | | | | | | | |
|  | **BASELINE** | | | **1 month** | | | **3 months** | | | **6 months** | | | **12 months** | | |
|  | control | test | *p** | control | test | *p** | control | test | *p** | control | test | *p** | control | test | *p** |
| **Bizzarro et al. 2017** | 87.86±9.62^†^ | 88.81±11.19^†^ | na | --- | --- | --- | 87.36±10.67^†^ | 85.06±11.44^†^ | na | 89.21±5.92^†^ | 85.43±10.28^†^ | na | 88.27±11.65^†^ | 85.44±10.04^†^ | na |
| *p*** |  |  |  |  |  |  | na | na |  | na | na |  | na | na |  |
| **Montero et al. 2020** | 84.1±11.2^†^ | 91.3±18.2^†^ | *0.143* | --- | --- | --- | 89.6±5.4^§^ | 84.8±3.8^§^ | *0.019* | 86.9±1.8^§^ | 81.8±2.8^§^ | *0.009* | --- | --- | --- |
| *p*** |  |  |  |  |  |  | *ns* | *ns* |  | *ns* | *ns* |  |  |  |  |
| **Doke et al. 2021** | 80.8±11.8^†^ | 79±10.6^†^ | na | 76.8±9.9^†^ | 74.5±9.5^†^ | *0.54* | 77.1±11.6^†^ | 75.9±8.8^†^ | *0.84* | --- | --- | --- | --- | --- | --- |
| *p*** |  |  |  | *0.016* | *0.004* |  | *0.020* | *0.073* |  |  |  |  |  |  |  |
| **Milanesi et al.2023** | 82.73±12.02^§^ | 81.45±10.71^§^ | *0.58* | --- | --- | --- | 83.01±12.22^§^ | 84.16±14.94^§^ | *0.60* | 84.40±15.66^§^ | 83.01±12.23^§^ | *0.58* | --- | --- | --- |
| *p*** |  |  |  |  |  |  | *0.99* | *0.15* |  | *0.66* | *0.59* |  |  |  |  |
| **WC (cm)** | | | | | | | | | | | | | | | |
|  | **BASELINE** | | | **1 month** | | | **3 months** | | | **6 months** | | | **12 months** | | |
|  | control | test | *p** | control | test | *p** | control | test | *p** | control | test | *p** | control | test | *p** |
| **Bizzarro et al. 2017** | 99.29 ±9.66^†^ | 99.00 ±9.40^†^ | na | --- | --- | --- | 100.86±10.99^†^ | 96.98±8.94^†^ | na | 99.50±10.66^†^ | 98.19±7.75^†^ | na | 100.23±12.20^†^ | 99.88±6.54^†^ | na |
| *p*** |  |  |  |  |  |  | na | na |  | na | na |  | na | na |  |
| **Montero et al. 2020** | 119.0±9.1^†^ | 120.1±18.4^†^ | *0.848* | --- | --- | --- | 119.2±2.7^§^ | 120.1±4.5^§^ | *0.527* | 119.2±2.7^§^ | 120.1±4.6^§^ | *0.115* | --- | --- | --- |
| *p*** |  |  |  |  |  |  | *ns* | *ns* |  | *ns* | *ns* |  |  |  |  |
| **Doke et al. 2021** | 98.4±9.2^†^ | 97.8±6.0^†^ | *0.727* | 96.8±9.1^†^ | 95.8±6.5^†^ | *0.57* | 96.1±9.4^†^ | 95.1±6.2^†^ | *0.67* | --- | --- | --- | --- | --- | --- |
| *p*** |  |  |  | *0.003* | *0.004* |  | *0.001* | *0.000* |  |  |  |  |  |  |  |
| **Milanesi et al.2023** | 101.42±16.72^§^ | 102.56±13.47^§^ | *0.90* | --- | --- | --- | 101.05±13.27^§^ | 102.69±15.37^§^ | *0.51* | 100.12±13.24^§^ | 100.66±13.42^§^ | *0.81* | --- | --- | --- |
| *p*** |  |  |  |  |  |  | *<0.01* | *0.99* |  | *0.0001* | *0.01* |  |  |  |  |

Abbreviations: DBP: diastolic blood pressure; CRP: c-reactive protein; FBG: fasting blood glucose; HbA1c: glycated hemoglobin; HDL-cholesterol: high-density lipoprotein cholesterol; na: not available; ns: not significant; SBP: systolic blood pressure: WC: waist circumference.

* *inter-group comparison*

*** intra-group comparison when compared to baseline*

^§^ *data reported as mean (± standard error)*

† *data reported as mean (± standard deviation)*
